# Thrombin-Activated Platelets Protect Vascular Endothelium against Tumor Cell Extravasation by Targeting Endothelial VCAM-1

**DOI:** 10.3390/ijms23073433

**Published:** 2022-03-22

**Authors:** Chiou-Mei Lee, Ming-Ling Chang, Ren-Hao Chen, Fan-Wen Chen, Jo-Chuan Liu, Shun-Li Kuo, Hsin-Hsin Peng

**Affiliations:** 1 Laboratory Animal Center, Chang Gung Memorial Hospital at Linkou, Taoyuan 33305, Taiwan; lcm5132@gmail.com (C.-M.L.); kim80230@cgmh.org.tw (R.-H.C.); 2 Liver Research Center, Division of Hepatology, Department of Gastroenterology and Hepatology, Chang Gung Memorial Hospital at Linkou, Taoyuan 33305, Taiwan; minglingc@cgmh.org.tw; 3 Department of Medicine, College of Medicine, Chang Gung University, Taoyuan 33302, Taiwan; 4 Department of Obstetrics and Gynecology, Chang Gung Memorial Hospital at Linkou, Taoyuan 33305, Taiwan; amanda24cfw@yahoo.com.tw; 5 Graduate Institute of Biomedical Sciences, College of Medicine, Chang Gung University, Taoyuan 33302, Taiwan; d000017699@cgu.edu.tw; 6 Division of Chinese Medicine Obstetrics and Gynecology, Department of Traditional Chinese Medicine, Chang Gung Memorial Hospital at Linkou, Taoyuan 33305, Taiwan; b9105001@cgmh.org.tw; 7 School of Traditional Chinese Medicine, Chang Gung University, Taoyuan 33302, Taiwan; 8 Graduate Institute of Clinical Medical Sciences, College of Medicine, Chang Gung University, Taoyuan 33302, Taiwan; 9 Center for Molecular and Clinical Immunology, Chang Gung University, Taoyuan 33302, Taiwan

**Keywords:** platelet, VCAM-1, permeability, angiogenesis, transendothelial migration, anti-tumor activity

## Abstract

When activated by thrombin, the platelets release their granular store of factors. These thrombin-activated platelets (TAPLT) have been shown to be capable of ameliorating pro-inflammatory processes. In this study, we tested if TAPLT could also protect the endothelium against tumor-related pro-inflammatory changes that promote angiogenesis and metastasis. Using endothelial cell (EC) models in vitro, we demonstrated that TAPLT protected EC against tumor conditioned medium (TCM)-induced increases of reactive oxygen species (ROS) production, EC permeability and angiogenesis, and inhibited transendothelial migration that was critical for cancer cell extravasation and metastasis. In vivo observations of TAPLT-mediated inhibition of angiogenesis and pulmonary colonization in a BALB/c nude mouse model were consistent with the in vitro findings. Neutralization of vascular cell adhesion molecule-1 (VCAM-1) binding significantly inhibited the ability of TAPLT to interact with EC and abrogated the TAPLT-mediated protection of EC against tumor angiogenesis and metastasis. Taken together, these findings suggest that VCAM-1-mediated linkage to EC is required for TAPLT to confer protection of EC against tumor-induced permeation and angiogenesis, thereby resisting tumor extravasation and metastasis.

## 1. Introduction

The ability of tumor cells to alter the host endothelium is central to tumor angiogenesis and malignant progression. In the inflammatory tumoral microenvironment, tumor cells and host cells including endothelial cells (EC), leukocytes and platelets are in an active crosstalk that influences the angiogenesis and metastatic processes [1,2,3,4]. It has been proposed that the up-regulation of endothelial adhesion molecules on inflamed endothelium facilitates interactions between the circulating tumor cells and EC and enable the process of extravasation and metastasis via formation of strong cell–cell bonds, with the active involvement of immune cells [5,6,7,8,9,10].

Platelets play active and diverse roles in modulating cancer-related inflammatory responses via its ability to interact with both the immune cells and the endothelial cells [3]. With the activation-induced expression of myriad surface adhesion molecules that enable interaction with both endothelium and other cells in the vascular microenvironment, platelets possess mechanisms to alter endothelial permeability and expression of endothelial adhesion proteins in favor of tumor angiogenesis and metastasis [4,11,12,13,14]. On the other hand, platelet activation has also been linked to inflammatory resolution, vascular protection, wound healing, and inhibition of tumor cell activities including survival, proliferation progression, and vasculogenic mimicry [15,16,17,18,19,20]. Papa et al. reported that detergent-treated human platelets can inhibit the development of thrombosis and metastasis [21]. The potential of platelets to render protection to the endothelium against tumor cell invasion may be attributed to the receptor–ligand interaction between the platelets and the endothelial cells, which could trigger secretion of soluble granular factors that promote endothelial integrity, even as the platelet itself directly plugs the breech in endothelial layer [22,23]. When activated, platelets exocytose granular vesicles; fusion of the exocytosed vesicles to the plasma membrane could potentially modify the profile of adhesion molecules presented at the surface of the platelets [24,25]. These adhesion molecules and receptors, presented on the surface of activated platelets, can interact with the other platelets and leukocytes as well as with the endothelium to mediate immune responses [26,27]. Platelet-derived thrombin is released into blood stream by diffusion, and the activity of thrombin in blood is modulated by plasma-born factors, leading to gradient-dependent heterogeneity in platelet activity, shape, size, density, metabolism, and binding capacity of coagulation factors [28,29,30]. The resulting circulating heterogeneous platelets may have roles in physiological and pathological processes beyond hemostasis and thrombosis [30,31]. It is reported that primed and isotonic buffer-washed human platelets had prolonged aggregation activity when challenged again [32,33]. Nevertheless, despite the wealth of knowledge of the multifaceted aspects of platelet involvement in tumor angiogenesis, metastasis, and maintenance of endothelial integrity, much remains to be learned on the exact nature of the platelet-mediated effects via study of platelets in their various states of post-activation changes.

Here, we demonstrate the protective role of thrombin-activated platelets (TAPLT) to EC with potent anti-angiogenesis and anti-metastatic effects. The effect of TAPLT on EC was first shown in a cell model, followed by validation in a relevant animal model. We also report the novel finding that the protective effect of TAPLT is achieved by direct TAPLT-endothelium interaction via vascular cell adhesion molecule 1 (VCAM-1) on endothelium. The involvement of VCAM-1 in TAPLT-mediated suppression of endothelial angiogenesis and permeability presents a new avenue of therapeutic approaches against tumor metastasis.

## 2. Results

### 2.1. Thrombin-Activated Platelets (TAPLT) Suppress Tumor Conditioned Medium (TCM)-Induced Endothelial Reactive Oxygen Species (ROS) Production

**Figure 1:**
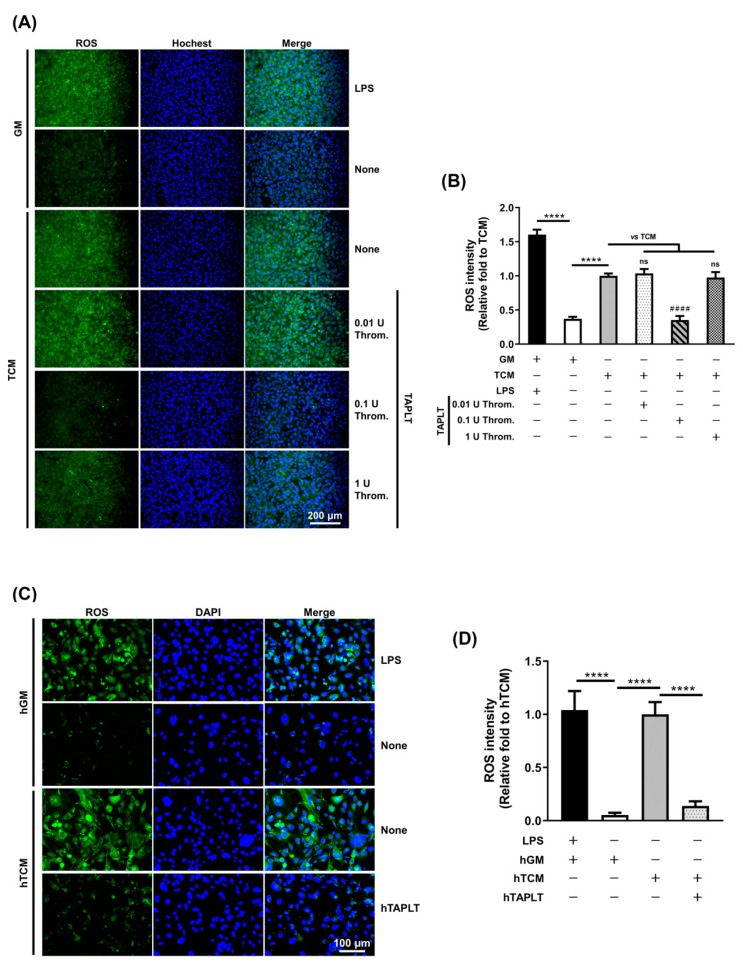
Thrombin-activated platelets (TAPLT) inhibit tumor-induced reactive oxygen species (ROS) production. (**A**) TAPLT were added to tumor conditioned medium (TCM)-treated murine endothelial cells, 2H11; ROS production was visualized by Rhodamin123 (green). Lipopolysaccharide (LPS) was used as positive control. (**B**) The ROS production seen in (**A**) was quantitated by fluorescence intensity. Data represent the total of three independent experiments with three fields per sample in each experiment. ****: *p* < 0.0001 by two-group comparison (*t*-test). ####: *p* < 0.0001 by multiple comparisons (ANOVA) to TCM treatment (“TCM”) as control; ns: not significant. (**C**) TAPLT derived from human platelets (hTAPLT) were applied to human TCM (hTCM)-treated human dermal microvascular endothelial cells, HMEC-1, and visualized for ROS production. (**D**) The ROS production seen in (**C**) was quantitated by fluorescence intensity. Data represent the total of three independent experiments with three fields per sample in each experiment. ****: *p* < 0.0001 by *t*-test.

We hypothesized that platelets in their various states of activation could contain a subset with protective activity on the endothelium. Therefore, we generated three subsets of mouse TAPLT using different doses of thrombin (0.01 U, 0.1 U, or 1 U) for treatment. We examined the effects of TAPLT on EC in response to TCM-induced oxidative stress by determining ROS levels. Treatment of the *Mus musculus* (mouse) endothelial cell line, 2H11 EC, with TCM resulted in a significant induction of ROS expression, as visualized by the increase in the intensity of green fluorescence, versus cells treated with control growth medium (Figure 1A,B). Of the three subsets of mouse TAPLT tested, the 0.1 U thrombin-treated TAPLT yielded the most significant reduction of TCM-induced endothelial ROS production (Figure 1A,B). Moreover, when treated with 0.1 U thrombin-derived human TAPLT (hTAPLT), the human dermal microvascular endothelial cells (HMEC-1) exhibited a significant reduction of intracellular ROS levels induced by TCM derived from the culture of human ovarian adenocarcinoma cells (SKOV3; Figure 1C,D). HMEC-1 was selected for the in vitro study for the functional and phenotypic characteristics of these cells, which resemble primary human microvascular endothelial cells [34], with the goal being validation of their findings in vivo. SKOV3 was used in our studies for its well-established use in the studies of tumor malignancy and angiogenesis [14].

### 2.2. TAPLT Suppress TCM-Induced EC Permeability

**Figure 2:**
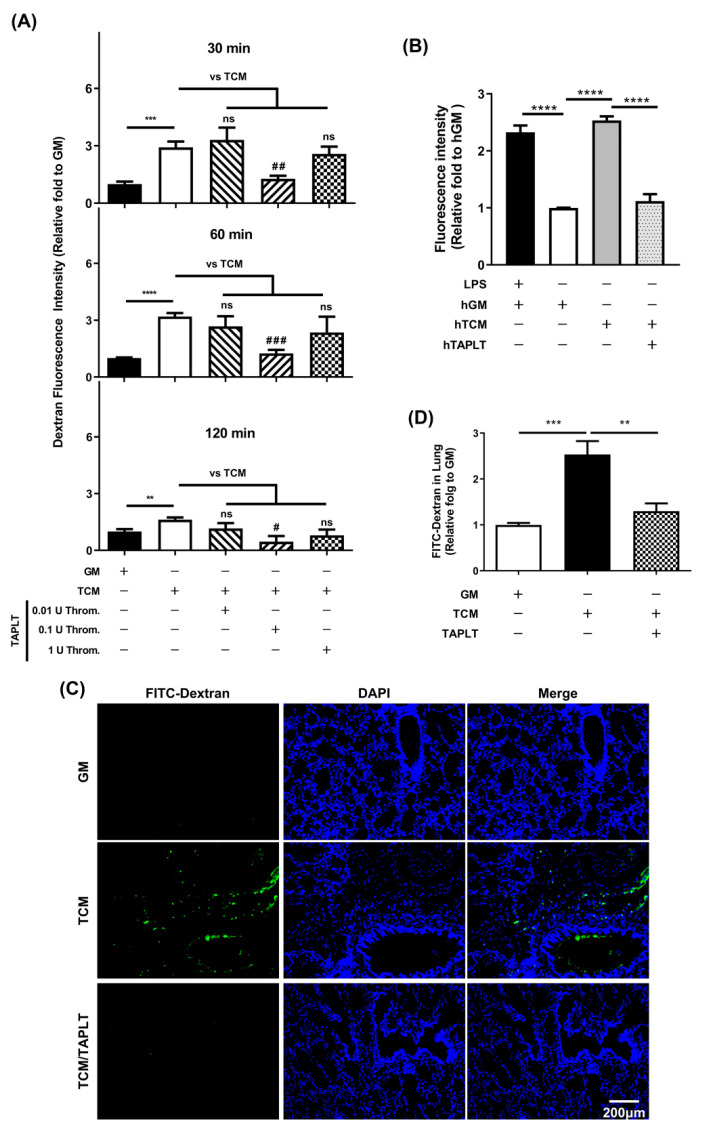
TAPLT ameliorate tumor-driven endothelial permeation. (**A**) TAPLT generated by 0.01 U, 0.1 U, or 1 U thrombin were co-cultured with TCM-treated 2H11 EC for the length of time indicated. Endothelial permeability relative to growth medium (GM) treatment was quantitated by dextran fluorescence intensity. Mean ± SEM, with a total of at least four individual experiments. **: *p* < 0.01; ***: *p* < 0.001; and ****: *p* < 0.0001 by *t*-test. #: *p* < 0.05; ##: *p* < 0.01; and ###: *p* < 0.001 by ANOVA with TCM treatment alone (“TCM”) as control. ns: not significant. (**B**) Human TAPLT (hTAPLT) were added to TCM-treated human dermal microvascular endothelial cells, HMEC-1, in the same assay setting. Mean ± SEM, with a total of three individual experiments. ****: *p* < 0.0001 by *t*-test. (**C**) Endothelial permeation in the lung of BALB/c nude mice was visualized by FITC-dextran signals. The extent of permeation was quantitated for comparison in (**D**). Each column represents 8 animals. Mean ± SEM; **: *p* < 0.01; and ***: *p* < 0.001 by *t*-test.

Endothelial ROS production in response to mouse TCM treatment during tumor progression elicits endothelial dysfunctions that include increased endothelial permeability. We thus added the mouse TAPLT derived from 0.01 U, 0.1 U, or 1 U thrombin treatment to 2H11 EC monolayer concurrent with TCM stimulation to measure the changes of EC permeability at 30, 60, and 120 min post TCM stimulation. The TCM-induced increases in the permeability of 2H11 EC monolayer with a significant induction was observed at 30, 60, and 120 min post stimulation, as compared to respective control growth medium (Figure 2A). Consistent with the observed suppression of TCM-induced endothelial ROS production, the 0.1 U thrombin-derived TAPLT achieved the greatest abrogation of the increase in EC permeability of 2H11 cell monolayer at all time points tested (Figure 2A). Our results suggest that the 0.1 U thrombin-derived TAPLT could exert the greatest protective effect on EC than the TAPLT derived from activation by the higher and lower doses of thrombin. Moreover, treatment of the HMEC-1 monolayer with human TAPLT (hTAPLT) derived from degranulation by thrombin led to a significant reduction of endothelial permeability (Figure 2B).

Since vascularization of the lung provided an efficient model for studying tumor cell metastatic dissemination, we used the model to study the effects of TAPLT on TCM-induced pulmonary hyperpermeability in vivo. In BALB/c nude mice intravenously injected with either TCM or TCM with TAPLT in suspension (TCM/TAPLT), pulmonary hyperpermeability was visualized with a fluorescein-5-isothiocyanate (FITC)-dextran tracer, followed by immunofluorescence staining. Compared with the control growth medium (GM) group, the pulmonary FITC-Dextran infiltration was diffused in TCM-inoculated mice; co-administration of TAPLT abrogated TCM-mediated endothelial permeation (Figure 2C). Moreover, co-administration of TAPLT with TCM effectively suppressed TCM-mediated exudation of FITC-dextran in lung lysates, as determined by quantitative analysis of fluorescence (Figure 2D). These results suggest that TAPLT are sufficient to inhibit the injurious effects of TCM on pulmonary endothelial barrier function.

### 2.3. TAPLT Suppress Tumor-Associated Angiogenesis

**Figure 3:**
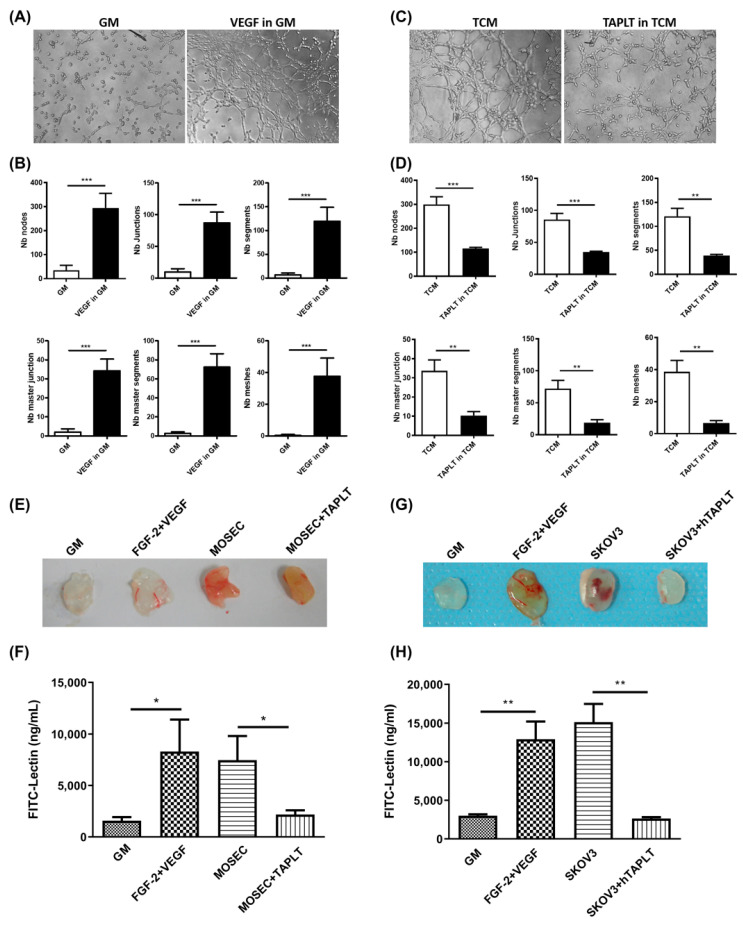
TAPLT inhibit tumor-associated angiogenesis. (**A**) Mouth endothelial cells, 2H11 EC, formed the tube network in solidified Matrigel at 2-h growth in GM in presence or absence of vascular endothelial growth factor (VEGF), respectively used as positive controls and negative controls. Representative images at 100× magnification were shown. (**B**) Quantitation of panel (**A**) using Image J with the Angiogenesis Analyzer plug-in tool. Mean ± SEM, with a total of three individual experiments. ***: *p* < 0.001. (**C**) Similar to control panel (**A**), the tube network formation of 2H11 EC in TCM in presence or absence of mouse TAPLT was studied. (**D**) Quantitation of panel (**C**). Mean ± SEM, with a total three individual experiments; **: *p* < 0.01; ***: *p* < 0.001. (**E**) The effect of GM alone (“GM”), GM with fibroblast growth factor 2 (FGF-2) and VEGF (“FGF-2+VEGF”), C57BL/6 murine ovarian surface endothelial cells (MOSEC; “MOSEC”), or MOSEC cells co-administered with TAPLT (“MOSEC+TAPLT”) on neovascularization of in vivo Matrigel plugs was imaged using digital camera. (**F**) Matrigel plugs observed in panel (**E**) was digested for FITC-lectin staining of endothelial cells. The concentration of FITC-Lectin was calculated for neovascular quantitation. Mean ± SEM (n = 9, 8, 12, and 11 respectively for “GM”, “FGF-2+VEGF”, “MOSEC”, and “MOSEC+TAPLT”). *: *p* < 0.05. (**G**) Similar to panel (**E**), the effect of GM, FGF-2+VEGF, and human TAPLT+SKOV3 (human ovarian adenocarcinoma cells) on neovascularization was recorded by images. (**H**) Quantitation of neovascularization in Matrigel plugs of panel (**G**). Mean ± SEM (n = 4 for all conditions). **: *p* < 0.01. All comparisons were two-group comparisons (*t*-test) as indicated.

Since elevated ROS expression could also induce EC angiogenesis for tumor growth and progression [35], we assessed the effects of TAPLT on tumor neovascularization by in vitro observation of endothelial tube formation and EC migration. When incubated on Matrigel with the addition of positive controls of vascular endothelial growth factor (VEGF) in GM for 2 h, 2H11 EC showed a significant increase of capillary-like tube formation, in contrast to treatment with negative controls (Figure 3A,B). TCM-induced endothelial tube formation was significantly abrogated in the presence of TAPLT (Figure 3C,D).

We next determined the effects of TAPLT on in vivo angiogenesis, using the Matrigel plug assay in an animal model of subcutaneous implantation in BALB/c nude mice. Examination of the implanted Matrigel matrix supplemented with positive control of VEGF and fibroblast growth factor 2 (FGF-2) in mice showed a significant blood vessel formation on the 10th day, in contrast to controls of GM (Figure 3E–H). Marked blood vessel formation in the Matrigel plug was observed in animals injected with C57BL/6 murine ovarian surface epithelial cells (MOSEC); the formation was significantly abrogated by co-administration with murine TAPLT (Figure 3E,F). Similarly, SKOV3-induced blood vessel formation in plug was also significantly reduced by the presence of human TAPLT (Figure 3G,H). These findings suggest that TAPLT treatment inhibits tumor-associated angiogenesis.

### 2.4. TAPLT Suppress Tumor Cell Transendothelial Migration and Lung Metastasis

**Figure 4:**
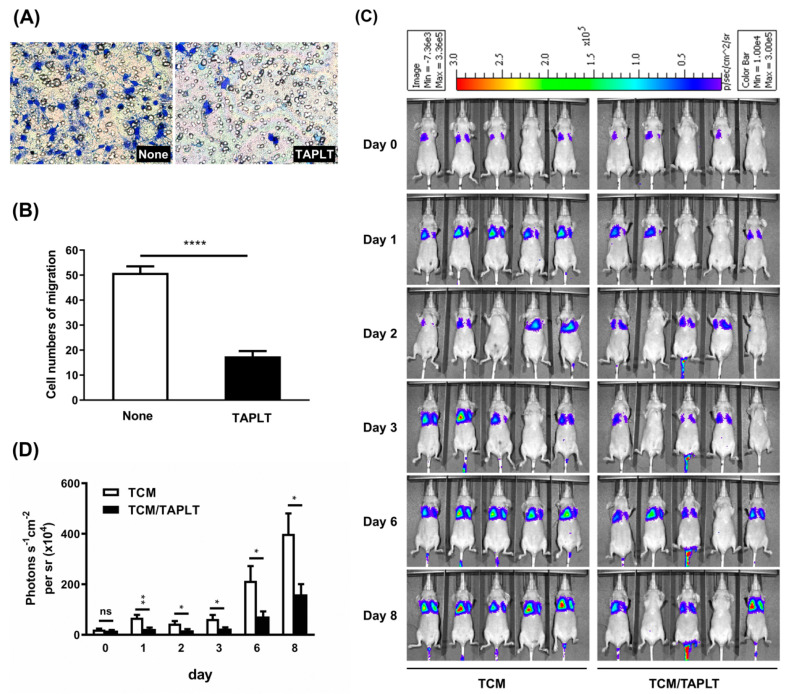
TAPLT inhibit tumor cell migration and metastasis. (**A**) MOSEC were co-cultured with TAPLT in a transendothelial migration assay through a 2H11 EC monolayer. Migrated cells were stained blue with DAPI. Representative images at 200× magnification were shown. (**B**) Number of migrated MOSEC was counted. Mean ± SEM; ****: *p* < 0.0001. (**C**) Representative images of in vivo pulmonary colonization assay of MOSEC in BALB/c nude mice without TAPLT co-administration (“TCM”) or with TAPLT co-administration (TCM plus TAPLT or “TCM/TAPLT” in figure). Lung metastatic foci were detected by IVIS (In Vivo Imaging System). Each animal was imaged on Day 0, Day 1, Day 2, Day 3, Day 6, and Day 8 for photon fluxes produced by Luciferase-tagged MOSEC (MOSEC/Luc). (**D**) Bioluminescent signals of the pulmonary colonization assay seen in (**C**) were quantitated (n = 18 for Day 0 to Day 6; n = 10 for Day 8). Mean ± SEM; *: *p* < 0.05, and **: *p* < 0.001; ns: not significant. All comparisons were two-group comparisons (*t*-test) as indicated.

Increased EC permeability is a critical process during tumor cell metastasis via blood vessels [36,37,38]. We therefore assessed the effects of murine TAPLT on cancer cell transendothelial migration prior to the studies of a mouse model of lung metastasis. Assays of MOSEC transmigration across a 2H11 EC monolayer showed that the presence of TAPLT in TCM resulted in significantly lower count of migrating cell numbers in contrast to controls (Figure 4A,B). We next examined the effects of TAPLT on the colonization of the lung by MOSEC. Luciferase-expressing MOSEC (MOSEC/Luc) were pre-incubated in TCM or TCM plus TAPLT for 30 min, and the incubation mixtures were then injected into BALB/c nude mice via tail vein. IVIS imaging analyses of each group of mice were performed 15 min after injection (i.e., day 0) and on days 1, 2, 3, 6, and 8. Compared with the control TCM treatment, there was a significantly lower extent of MOSEC/Luc colonization of the lung in the mice injected with TCM plus TAPLT (Figure 4C,D). Quantitative analysis revealed a significant reduction in bioluminescence signals indicative of the lung metastasis in mice injected with MOSEC in TCM plus TAPLT, as opposed to those injected with MOSEC in TCM alone (Figure 4D). It seemed unlikely that the inhibitory effect of TAPLT on cancer cell lung colonization was caused by the variation of the starting cell inoculation number, given that there was no significant difference in initial lung colonization by cancer cells on Day 0 in mice injected with either TCM plus TAPLT suspension or TCM alone (Figure 4C,D). These findings suggest that TAPLT may play a critical role on suppressing cancer metastasis by inhibiting TCM-facilitated extravasation-promoting changes of the endothelium.

### 2.5. Expression of Vascular Cell Adhesion Molecule 1 (VCAM-1) on EC Surface Is Essential for Binding, Adhesion, and Protective Role of TAPLT under TCM Stimulation

**Figure 5:**
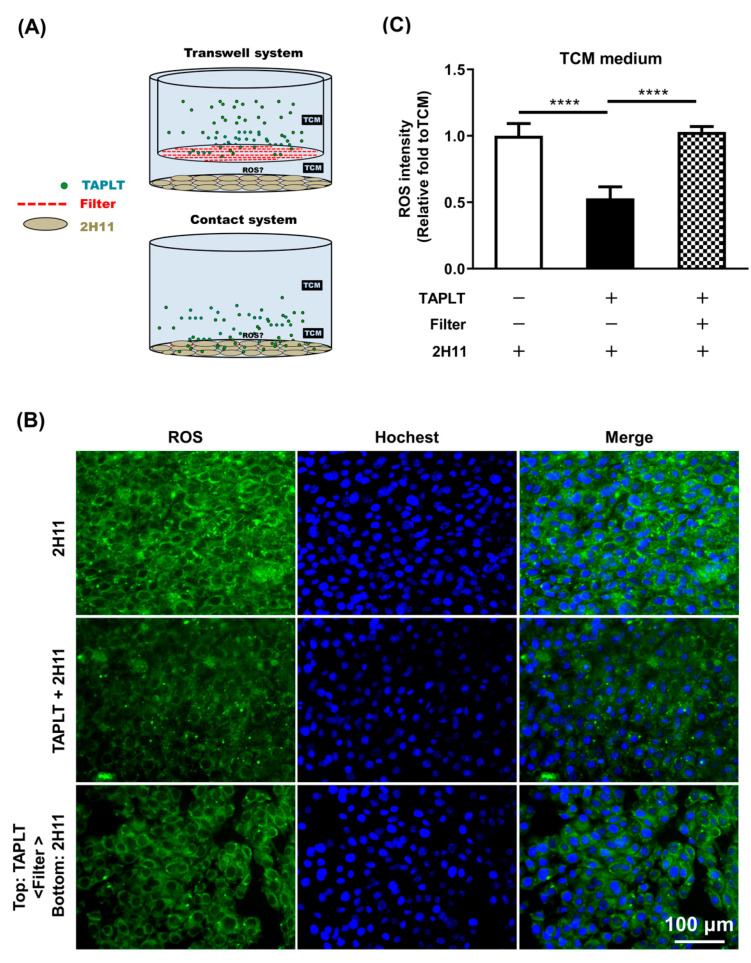
TAPLT directly interact with endothelial cells to render protection. (**A**) The Transwell system (top panel; pore size = 0.4 μm) and the contact system (bottom panel) were used to test indirect and direct TAPLT-2H11 EC interactions respectively. (**B**) Visualization of the cell co-culture by fluorescence microscopy at 200× magnification. Images are representative of at least three individual experiments. (**C**) The fluorescent intensity of ROS signals was quantified from three random fields for each sample. Data represent at least three individual experiments. Mean ± SEM; ****: *p* < 0.0001 by two-group comparisons (*t*-test) as indicated.

After confirming that TAPLT protected EC against TCM effects, we further investigated the requirement of direct cell–cell contact for the protective activity of TAPLT by Transwell co-culture of TAPLT and 2H11 EC in TCM (Figure 5A). Analysis of 2H11 EC revealed that TAPLT barred from physical contact with the endothelial monolayer in a Transwell co-culture failed to significantly impact the TCM-mediated elevation of endothelial ROS levels, whereas TAPLT in direct contact with the EC effectively suppressed TCM-mediated endothelial ROS production, showing a nearly complete reversion of ROS levels in comparison to TCM controls (Figure 5B,C). These results suggest that the direct cell–cell contact of TAPLT to 2H11 EC is required for TAPLT-mediated endothelial protection.

**Figure 6:**
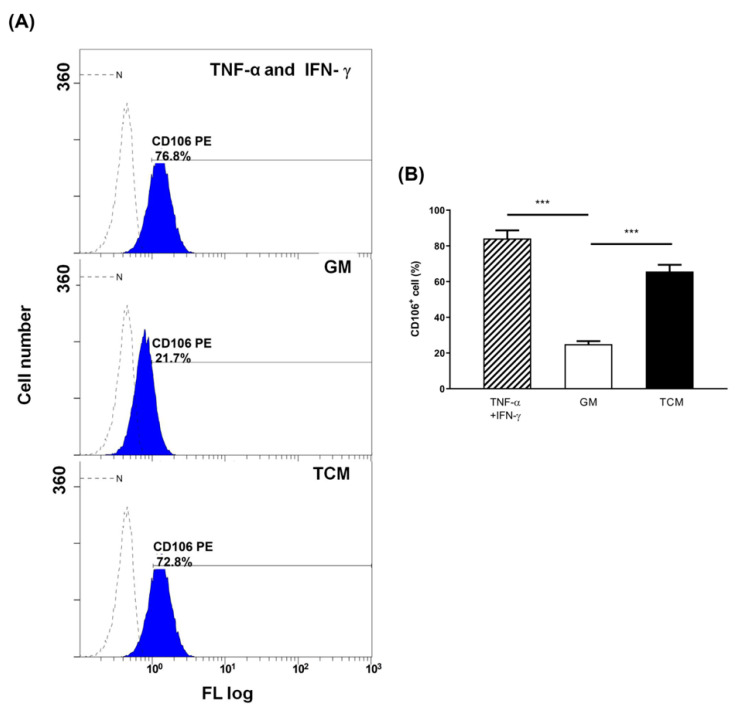
TCM increases endothelial VCAM-1 expression. VCAM-1 (CD106) on TCM-stimulated 2H11 EC were determined by flow cytometry. (**A**) Representative flow cytometry histograms of CD106 expression (blue peak) versus the corresponding isotype control (dashed line) on 2H11 EC. (**B**) Percentage of C106+ cells induced by TCM treatment (“TCM”) was compared to control cells in growth medium (“GM”) in three individual experiments. “TNF-α and IFN-γ”: cytokine treatment of tumor necrosis factor-α (TNF-α) and Interferon-γ (IFN-γ) as positive control to GM. Mean ± SEM; ***: *p* < 0.001 by *t*-test.

**Figure 7:**
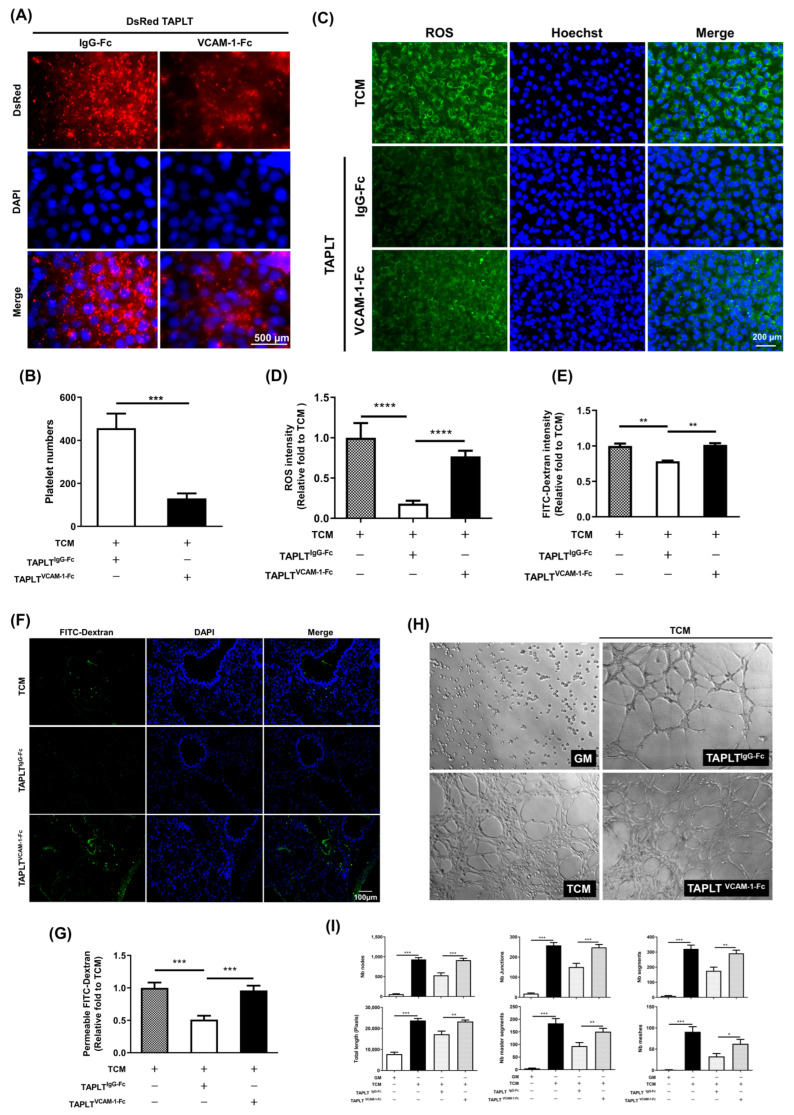
Neutralization of VCAM-1 binding abrogates TAPLT protection against tumor-mediated endothelial changes. (**A**) Recombinant VCAM-1-Fc chimera protein was added to co-culture of DsRed-tagged TAPLT on 2H11 EC monolayer. Images at 400× magnification. (**B**) The number of endothelial-adherent TAPLT in three random fields per sample was counted. TAPLT^VCAM-1-Fc^: TAPLT treated with VCAM-1-Fc. TAPLT^IgG-Fc^: TAPLT treated with IgG-Fc. Mean ± SEM; ***: *p* < 0.001. (**C**) ROS production in 2H11 endothelial monolayer was visualized with or without TAPLT co-incubated with VCAM-1-Fc chimera. (**D**) Data were quantified from a total of at least five individual experiments. Mean ±SEM; ****: *p* < 0.0001. (**E**) Endothelial permeability assay of confluent EC treated by TAPLT^VCAM-1-Fc^ or control TAPLT^IgG-Fc^ in TCM. Data represent three individual experiments. Mean ± SEM; **: *p* < 0.01. (**F**) Lung permeability assay of BALB/c nude mice injected with TCM suspended TAPLT^VCAM-1-Fc^ or control TAPLT^IgG-Fc^ in presence of FITC-Dextran. Representative images (100× magnification) were acquired from paraffin sections of lungs for FITC-Dextran signals (green). (**G**) Lung permeation seen in (**F**) was quantitated by FITC-Dextran intensity. Mean ± SEM (n = 6 for all conditions). ***: *p* < 0.001. (**H**) The tube network formation of 2H11 EC in TCM in presence of TAPLT^VCAM-1-Fc^ or control TAPLT^IgG-Fc^ was studied. Representative images at 100× magnification. (**I**) Quantitation of panel (**A**) using software Image J and the Angiogenesis Analyzer plug-in tool. Mean ± SEM, with a total of three individual experiments. *: *p* < 0.05; **: *p* < 0.01; ***: *p* < 0.001. All comparisons were two-group comparisons (*t*-test) as indicated.

Elucidating the molecular basis of TAPLT-cell contact is critical for understanding TAPLT protection of EC against TCM effects. We therefore performed a screening of adhesion proteins to identify those whose expression on the cellular surface of the 2H11 EC was affected by TCM. The surface expressions of integrin α (CD51), VCAM-1 (CD106), PSGL-1 (CD162), ICAM-1 (CD54), P-selectin (CD62P), PECAM-1 (CD31), and E-selectin (CD62E) were determined by flow cytometry analysis. Of the adhesion proteins screened, only VCAM-1 expression was markedly elevated in 2H11 EC at 4-h post stimulation with 50% TCM mixture (TCM:10% Fetal Bovine Serum-Rosewell Park Memorial Institute 1640 (FBS-RPMI) = 1:1, *v*/*v*; Figure 6A,B and Figure S1A,B). To evaluate the requirement of endothelial VCAM-1 for TAPLT-related effects, we analyzed whether VCAM-1 binding by TAPLT could be neutralized with a recombinant VCAM-1-IgG-Fc chimera protein (VCAM-1-Fc) to abrogate TAPLT-mediated protection against the impact of TCM on 2H11 EC. In contrast to the control treatment of TAPLT prehybridized with recombinant human IgG1 Fc (TAPLT^IgG-Fc^), TAPLT prehybridized with the VCAM-1-IgG-Fc chimera (TAPLT^VCAM-1-Fc^) lost their capacity to bind the 2H11 EC (Figure 7A,B). Similarly, TAPLT^IgG-Fc^, but not TAPLT^VCAM-1-Fc^, were capable of reducing ROS production by TCM-conditioned 2H11 EC (Figure 7C,D), and co-culture with TAPLT^IgG-Fc^, but not TAPLT^VCAM-1-Fc^, were effective of suppressing TCM-mediated endothelial permeability (Figure 7E). Consistent with this observation of in vitro permeability assay, TAPLT^IgG-Fc^, but not TAPLTV^CAM-1-Fc^, efficiently inhibited TCM-induced lung hyperpermeability (Figure 7F,G). Furthermore, TAPLT^IgG-Fc^, but not TAPLT^VCAM-1-Fc^, abrogated the ability of TCM-conditioned 2H11 EC to form capillary-like tube in a Matrigel (Figure 7H,I). Moreover, TAPLT were seen to selectively express the VCAM-1 counter-ligand αD/β2 integrin on its surface (Figure S2). Neutralizing anti-αD or anti-β2 integrin antibody significantly abrogated the effects of TAPLT against TCM-activated EC on their adhesion and permeability inhibition (Figures S3 and S4). These results suggest that the expression of αD/β2 integrin on the surface of the TAPLT confer protection to EC against tumor-induced activity by interacting with endothelial VCAM-1.

### 2.6. TAPLT-Mediated Modulation of Tumor Cell Transendothelial Migration and Metastasis Requires TAPLT Binding to Endothelial VCAM-1

**Figure 8:**
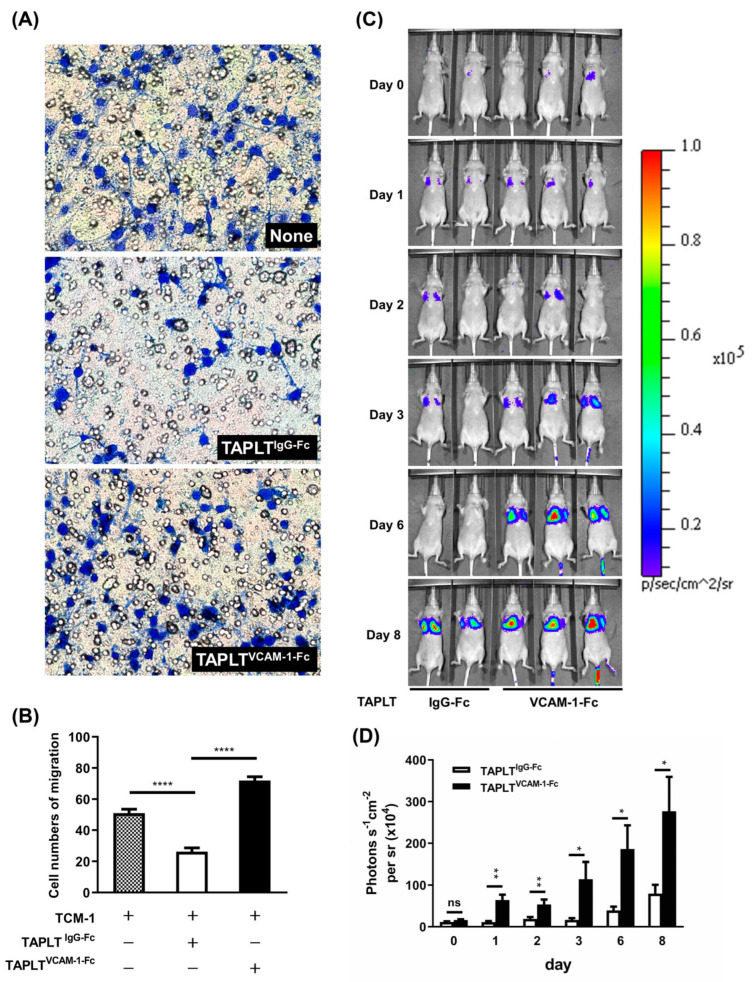
Neutralization of VCAM-1 binding abrogates TAPLT benefit on suppressing tumor migration and metastasis in vivo. (**A**) Transendothelial migration assay of TAPLT-co-cultured MOSEC cells on 2H11 monolayer in presence of VCAM-1-Fc chimera. Images (200× magnification) are representative of three individual experiments. (**B**) Migratory cells seen in (**A**) were counted from at least three random fields for each sample. Mean ± SEM; ****: *p* < 0.0001. (**C**) Representative images of in vivo pulmonary colonization assay of MOSEC in BALB/c nude mice co-administered with IgG-Fc-treated TAPLT (“TAPLT^IgG-Fc^”) or with VCAM-1-Fc-treated TAPLT (“TAPLT^VCAM-1-Fc^”). Lung metastatic foci were detected by IVIS imaging system. Each animal was imaged on Day 0, Day 1, Day 2, Day 3, Day 6, and Day 8 for photon fluxes produced by MOSEC/Luc. (**D**) Bioluminescent signals of the pulmonary colonization assay seen in (**C**) were quantitated (“TAPLT^IgG-Fc^”: n = 22 for Day 0 to Day 6; n = 14 for Day 8; “TAPLT^VCAM-1-Fc^”: n = 20 for Day 0 to Day 6; n = 16 for Day 8). *: *p* < 0.05; **: *p* < 0.01; ns: not significant. All comparisons were two-group comparisons (*t*-test) as indicated.

We further examined whether the effects of TAPLT on inhibition of tumor cell extravasation and metastasis involve the VCAM-1 binding. Assays of in vitro transendothelial cell migration showed the number of migrated cancer cells through 2H11 EC in response to TCM was reduced by TAPLT^IgG-Fc^, but not by TAPLT^VCAM-1-Fc^, in contact with the EC monolayer (Figure 8A,B). We also observed the impact of TAPLT^VCAM-1-Fc^ on lung metastatic colonization of TCM-stimulated cancer cells, in which TAPLT^IgG-Fc^, but not TAPLT^VCAM-1-Fc^, effectively suppressed TCM-mediated metastatic lung colonization by the MOSEC cancer cells (Figure 8C). Quantitative analysis of the images of in vivo metastatic lung colonization confirmed the findings that only TAPLT^IgG-Fc^ and not TAPLT^VCAM-1-Fc^ were capable of deterring experimental lung metastasis when injected into mice along with TCM, as detected by bioluminescence imaging signals within 1 to 8 days post injection (Figure 8D). Thus, the binding to endothelial adhesion molecule VCAM-1 by TAPLT is closely associated with their capacity of protecting EC against TCM-mediated permeation and pro-inflammatory activation that facilitate cancer metastasis through the endothelium.

## 3. Discussion

In this study, we demonstrated that TAPLT had a potent protective effect against TCM-induced EC angiogenesis and permeability, thereby exerting anti-tumor activity. The endothelial protection and anti-tumor activity of the particular subset of platelets facilitated through engagement of VCAM-1; neutralization of VCAM-1 couple ligand by competitive VCAM-1 abrogated the anti-tumor activity of these platelets. The TAPLT were derived in vitro by treatment with thrombin, one of the most potent platelet agonists, at an intermediate dose at 0.1 U. Studies demonstrated that 0.1 U thrombin-primed, washed, and disaggregated platelets significantly alleviated inflammatory responses in rates with acute respiratory distress syndrome/sepsis injury or acute kidney ischemia/reperfusion injury [39,40,41]. These washed platelets were deprived of as much as 90% of their dense granule and α-granule contents as a result of stimulation by thrombin [42]. We confirmed a dose–response relationship of platelet activation other than the release of soluble platelet factors, where typical levels of thrombin reaction may result in subpopulations of pro- or dis-aggregatory phenotypes of platelets with opposite regulatory functions on proinflammatory activation.

In the clinical context, cancer patients have abundant tissue factors that promote the conversion of prothrombin to thrombin [43]. The magnitude of thrombin release and reaction ultimately depends on the persistent concentration of clotting factors, tissue factor and circulating tumor cells at local and distributed site [44,45]. Due to the heterogeneous deposition of thrombin to the vascular endothelium, the uneven spatial distribution may result in differential surface density of thrombin molecules, leading to differentiated activity of platelets interacting with the vascular endothelium.

In vivo studies of vascular leakiness and lung metastatic colonization further confirmed that TCM could damage EC and increase cancer metastasis. The effect of TCM was mediated through induction of surface VCAM-1 expression by the EC, as demonstrated in this study. This observed effect of TCM was consistent with past findings of tumor cell activity of pro-metastasis modification of the vascular endothelium [36,37,38], which also involved pro-inflammatory processes such as production of Interleukin 8 (IL-8) and recruitment of immune components including neutrophils [46,47]. These observed effects, elicited by TCM, can be blocked by concurrent treatment with TAPLT that were primed with 0.1 U thrombin. This antioxidant and anti-inflammatory activities were mediated through inhibition of ROS production within EC. It has been shown that ROS production-inducing agents such as menadione cause oxidant stress within endothelial cells like HMEC-1, thus directly impacting their cellular viability [48]. Conversely, modulation of ROS production in endothelial cells by antioxidant mechanisms such as the induction of the nuclear factor (erythroid 2-related factor) 2 (Nrf2) reduces oxidative stress, promotes cellular viability and downregulation of proinflammatory expression of surface adhesion molecules, and restores endothelial function [49]. The ability of TAPLT to modulate endothelial ROS production is therefore of critical functional consequence that may lead to alleviation of endothelial dysfunctions that include the changes of endothelial permeability [50]. As passage of cancer cell through the endothelial barrier is required for further metastasis [51], this maintenance of endothelial integrity by the thrombin-activated platelets potentially prevents this passage from taking place

Our results showed that TAPLT bound directly to EC to actively inhibit TCM-induced endothelial ROS production. This direct contact between the platelets and the endothelium was facilitated by VCAM-1, as it was abrogated by competitive inhibition with soluble VCAM-1/CD106 Fc recombinant protein. The requirement of VCAM-1 for platelet interaction with the endothelium suggests a possible mechanism in which pro-inflammatory endothelial dysregulation, facilitated by TCM, in turn recruits the anti-tumor subsets of platelets capable of maintaining and repairing endothelial integrity. Akin to leukocyte presentation of integrin α4β1 (also named very late antigen 4, VLA-4), which is a principal ligand for VCAM-1, platelets express αD/β2 integrin, another ligand of VCAM-1 that enables platelets to specifically interact with this receptor [52]. As demonstrated in our findings, the αD/β2 integrin-VCAM-1 interaction between TAPLT and the endothelium regulates endothelial permeability, thereby impacting on transendothelial migration of tumor cells. Aberrant increase of VCAM-1 expression has been implicated in various diseases with acute or chronic inflammation [53]. Although the effect of increased VCAM-1 expression on EC is more prominent in the retention and transmigration of leukocytes during inflammatory or neoangiogenic process, it is known that tumor cell adhesion and metastasis were also affected, as these cells express VCAM-1-binding ligands such as VLA-4 and secreted protein acidic and rich in cysteine (SPARC) [54,55]. In this context, the involvement of VCAM-1 in both pro-inflammatory and pro-metastasis processes provides an entry point of anti-tumor activity through endothelial modulation.

Day et al. suggested that thrombin-primed, shape-changed platelets in their pre-activation and disaggregated state could have anti-inflammatory activity in acute injuries, and that these anti-inflammatory effects could be attributed to a decrease in leukocyte infiltration [41]. Flow cytometry analysis of platelets treated with thrombin revealed that they were indeed in a state of activation, featuring the increase of platelet activation marker P-selectin (CD62P) expression in response to thrombin treatment at 0.1 U and above (Figure S5A,B). Scanning electron microscopy (SEM) observations of the increasing platelet shape change responses (Figure S6) confirmed an increase in intensity of platelet activation following their exposure to the increasing dose levels of thrombin. The present findings therefore identify a subset of platelet activity that is specific to activating condition and its involvement in modulating the normal endothelial function and its potential to prevent cancer metastasis. Although the disaggregates or microaggregates of activated platelets in their physiological state are labile towards agonistic aggregation and eventual coagulation [56], it is technically feasible to boost the particular anti-cancer subset to a therapeutically meaningful extent. This was demonstrated in our study of the potent anti-cancer capacity of thrombin-activated platelets, which had heretofore been unobserved. While these subset-specific observations appear to contrast with the past clinical observations of malignance-associated activation of platelets in terminal cancer patients with hypercoagulability featuring massive thrombosis [57,58,59,60,61], this contradiction may be due to the non-specific physiological activation of platelets masking the anti-cancer aspect of this particular subset of platelets. Indeed, TAPLT exhibited diminished aggregation reactivity versus naïve platelets in response to thrombin/fibrinogen induction, as demonstrated in an analysis of differential activity of platelets (Figure S7A). Since aggregation of platelets with involvement of tumor cell has been implicated in promoting tumor invasion, proliferation, and metastasis [1,62,63], this loss of aggregation capacity in platelets is suggestive of modified platelet functions that may potentially impact cancer cell activities. Moreover, the thrombin-activated platelets have relatively little effect on the aggregation of untreated, naïve platelets in response to thrombin/fibrinogen induction (Figure S7B). With the possibility of selectively generating the anti-tumor platelet subset with minimal downside effect, as opposed to anti-platelet agents such as heparin and aspirin, which have an increased risk of hemorrhage [64], TAPLT may emerge as a safer therapeutic option against metastasis.

In conclusion, our study on the interaction between TAPLT and endothelium identified a novel mechanism of TAPLT-mediated, VCAM-1-dependent endothelial protection against tumor-associated damages. Our findings on the specific post-activation subset of platelets that actively promoted endothelial integrity via VCAM-1 presentation provides a perspective on platelet-mediated anti-tumor effect that to our knowledge has not been fully explored previously. Understanding the protective platelet–endothelium interaction may provide new insights on how platelets confer a negative effect on tumor metastasis and angiogenesis, with implications for potential therapeutic interventions against cancer, such as cell therapy against tumor angiogenesis or metastasis with TAPLT derived from the patient’s own platelets.

## 4. Materials and Methods

### 4.1. Ethics Approval of Human Subjects

The protocol of all studies involving human subjects was approved by the Institutional Review Board of Chang Gung Memorial Hospital (CGMH), Taiwan as described in the Institutional Review Board Statement.

### 4.2. Animals

C57BL/6C mice and BALB/c nude mice were purchased from Charles River Taiwan Branch (female, 8 weeks of age, BIOLASCO LTD, Taipei, Taiwan). The C57BL/6C mice were used for the isolation of platelets, and the BALB/c nude mice were used for all in vivo studies. In addition, C57BL/6J-Tg (UBC-DsRed-emGFP) 22Narl (RMRC13119) mice were acquired from the National Laboratory Animal Center (NLAC, Taipei, Taiwan) and used for the preparation of DsRed-tagged TAPLT. The protocols of all studies involving animals were approved by the IACUC of CGMH as described in the Institutional Review Board Statement.

### 4.3. Cell Lines

The C57BL/6 mouse ovarian surface epithelial cells (MOSEC) and luciferase-expressing MOSEC tumor cell line (MOSEC/Luc) were generously provided by Dr. Tzu-Hao Wang and were maintained in RPMI 1640 media supplemented with 10% FBS (Gibco, Thermo Fisher Scientific, Waltham, MA, USA), 1 mM sodium pyruvate, 1× Gibco^®^ MEM non-essential amino acid, 2 mM glutamine, 0.2 mM β-mercaptoethanol. In addition to MOSEC and MOSEC/Luc cells, all other cells were acquired from the American Type Culture Collection (ATCC, Manassas, VA, USA) for this study. The mouse endothelial cell line 2H11 was maintained in DMEM media supplemented with 10% FBS and 0.405 g/L glucose. The human ovarian cancer cell line SKOV3 was maintained in McCoy’s 5A Medium (30-2007; ATCC) supplemented with 10% FBS. The human endothelial cell line HMEC-1 was maintained in MCDB131 media supplemented with 10% FBS, 10 ng/mL Epidermal Growth Factor (EGF), 1 µg/mL Hydrocortisone, and 10 mM Glutamine. All cells were cultured under full humidification at 5% CO_2_ and 37 °C.

### 4.4. Preparation of Tumor Conditioned Medium

For mouse cancer cell-derived tumor conditioned medium (TCM), 1 × 10^6^ MOSEC ovarian cancer cells were seeded in 10-cm dishes for 2-day culture in 10 mL of 10% FBS/RPMI 1640 medium (Thermo Fisher Scientific, Waltham, MA, USA), and the cell free medium was collected by centrifugation (200× *g*, 5 min) for further experiments. For human cancer cell-derived TCM (hTCM), 5 × 10^5^ SKOV3 ovarian cancer cells (HTB-77; ATCC, Rockville, MD, USA) were seeded in 10-cm dishes for 3-day culture in 10 mL of 10% FBS/McCoy’s 5a medium, and cell free medium was collected by centrifugation (200× *g*, 5 min) for further experiments.

### 4.5. Preparation of Platelets and TAPLT

Mouse or human blood was collected in a sterile tube containing the citrate-dextrose solution (ACD; Sigma, St. Louis, MO, USA) as anti-coagulant, with the volume ratio of blood to ACD at 1:6 (mouse) or at 1:9 (human). The ACD-mixed whole blood was divided into 5 mL per tube, and then centrifuged at 25 °C, 2300× *g* for 40 s (mouse) or for 30 s (human) at maximum acceleration and no brake at deceleration. The upper layer containing platelet-rich plasma (PRP) was transferred to a fresh tube, and its volume was adjusted to 5 mL containing Heparin (50 U) and PGI2 (2.5 nM; Sigma, St. Louis, MO, USA). This PRP suspension was centrifuged at 25 °C, 2200× *g* for 10 min (mouse) or for 13 min (human) at maximum acceleration with no brake at deceleration. The supernatant was discarded, and the pellets were resuspended in 5 mL of Tyrode’s albumin buffer containing PGI2 (0.5 μM), and centrifuged at 2050× *g* (mouse) or at 1065× *g* (human) for 6 min at maximum acceleration with no brake at deceleration. The resulting pellets were next washed again with the same procedures, pelleted again, and then resuspended in Tyrode’s albumin buffer with apyrase (0.02 U/mL; Sigma, St. Louis, MO, USA). Platelet number was counted by a Drew Hemavet 950FS^®^ analyzer (Drew Scientific, Miami Lakes, FL, USA).

For TAPLT preparation, 0.01 U, 0.1 U, or 1 U thrombin (Sigma, St. Louis, MO, USA) was added to 0.5 mL of platelet suspension containing 2.5 × 10^8^ to 3 × 10^8^ platelets, and the transition of shape-changed platelets was tracked by the 560CA Whole Blood Lumi Aggregometer Aggregation System (Chrono-log Corp., Havertown, PA, USA), followed by adding hirudin (Sigma, St. Louis, MO, USA) to match the corresponding thrombin content units to stop the reaction. This resulting TAPLT suspension was centrifuged at 25 °C, 1300× *g* for 3 min, and then resuspended in Tyrode’s albumin buffer or experimental medium. In subsequent experimentations, TAPLT refer to the 0.1 U thrombin-derived platelet subset unless otherwise specified.

### 4.6. ROS Assay

An amount of 1 × 10^5^ 2H11 EC (CRL-2163; ATCC) were cultured on 50 μg/mL collagen Type I (BD Biosciences, San Jose, CA, USA)-coated glass coverslips in 10% FBS/DMEM medium and allowed to expand to confluence. After washing, the cells on the coverslip were incubated with or without mouse TAPLT (1 × 10^8^) in 1 mL of TCM, or in 10% FBS/RPMI 1640 medium in presence or absence of lipopolysaccharide (LPS, 5 μg/mL; Sigma, St. Louis, MO, USA), as positive or negative control, respectively, for 30 min in the presence of dihydrorhodamine-123 (DHR, 30 μM; Sigma, St. Louis, MO, USA). Post-incubation, Hoechst 33342 (20 mM; Thermo Fisher Scientific, Waltham, MA, USA) was added to the cultures for another 30 min incubation. The induced green fluorescence of Rhodamine 123, resulting from the oxidation reaction of DHR and H_2_O_2_ in cells, was captured by fluorescent microscopy (Axiovert 200M; Carl Zeiss Jena GmbH, Jena, Germany).

The procedures for the ROS assay and quantitation of HMEC-1 EC were similar to the above. In addition to the use of HMEC-1 EC instead of 2H11 EC, human TAPLT (1 × 10^7^/culture) and hTCM were used in the culture, and 10% FBS/McCoy’s 5a was used to as negative control medium. Moreover, ROS assays of HMEC-1 with LPS (5 μg/mL; Sigma) in 10% FBS/McCoy’s 5a growth medium (hGM) were used as positive control.

### 4.7. In Vitro EC Permeability Assay

2H11 mouse EC were seeded on the collagen (50 μg/mL; Sigma, St. Louis, MO, USA)-pre-coated upper-chamber Transwell filters of a 24-well Boyden Chamber system (0.4 μm pore size; Corning Costar, Santa Barbara, CA, USA) in 200 μL of 10% FBS/RPMI 1640 medium and allowed to grow to confluence. The upper chamber with confluent cell monolayer was then inserted to the lower-chamber well containing 800 μL of phenol red-free 10% FBS/RPMI 1640 medium. The top chamber with the monolayer was filled with 200 μL of either tumor conditional medium (TCM; phenol red free) with or without mouse TAPLT (1 × 10^8^), or control medium of 10% FBS/RPMI 1640 containing FITC-Dextran (0.5 mg/mL; MW = 70 kDa; Sigma, St. Louis, MO, USA). EC permeability was determined by measuring the passage of FITC-labelled dextran through the EC monolayer at 30, 60, or 120 min. The fluorescence of permeable FITC-Dextran was measured at the excitation wavelength of 485 nm and the emission wavelength of 530 nm using the Tecan Infinite M200 plate reader. Similar procedures were performed for the permeability assay of human endothelial cells HMEC-1, with HMEC-1 cells seeded and allowed to proliferate on the membrane of a Transwell upper chamber. The confluent EC monolayer was next incubated with or without human TAPLT (1 × 10^7^) in SKOV3-derived TCM for 60 min. The treatments of human HMEC-1 cells with 10% FBS/McCoy’s 5a growth medium with or without LPS (5 μg/mL; Sigma, St. Louis, MO, USA) were used as positive control and negative control, respectively.

### 4.8. Lung Vascular Permeability Assay

Female BALB/c nude mice were intravenously injected at the by tail vein with 100 μL of TCM with or without TAPLT (1 × 10^8^) as indicated at once daily for 2 consecutive days. Injection was performed with the animal in a restraining device. At 24 h after the second inoculation, FITC-Dextran (MW = 70 kDa; Sigma, St. Louis, MO, USA) was intravenously injected for 30 min at 1 mg per ~20 g of body weight per mice. The lung was removed from the animal for subsequent experimentation in a non-survival surgical procedure, in which the animal was subjected to deep-stage anesthesia by intramuscular injection of 150 μL of a mixture of Zoletil^®^ 50 (Virbac, Carros, France) and Rompun^®^ (2% Xylazine; Horse & Camel Supplies, Abu Dhabi, UAE) at a 1:1 *v*/*v* ratio, followed by the perfusion and then removal of the lung while under anesthesia. The extracted lungs were subjected to FITC-Dextran staining and measurement to detect permeability. The experimental conductions were performed by observations of immunofluorescence staining from lung sections and measurements of fluorescent intensity from lung lysates. For histological studies of permeable FITC-Dextran, 4μm of tissue sections were first incubated with goat anti-FITC antibody (1:100; Thermo Fisher Scientific, Waltham, MA, USA), followed by incubation with donkey anti-goat IgG conjugated with FITC of secondary antibody (1:100; Thermo Fisher Scientific, Waltham, MA, USA). DAPI was used for a nuclear counter stain. After sample mounting with antifade media, the extravasation of FITC-Dextran was examined by fluorescence microscopy (Zeiss Axioskop; Carl Zeiss, Oberkochen, Germany), and images were captured by digital camera. For measurements of permeable FITC-Dextran in lung lysates, lungs were homogenized in RIPA buffer, and then centrifuged to remove tissue debris. The fluorescence of the supernatant at 500 μg protein per sample was then measured at an excitation wavelength of 485 nm and an emission wavelength of 530 nm. Tissue content of FITC-Dextran (Thermo Fisher Scientific, Waltham, MA, USA) was calculated from a standard curve.

### 4.9. Endothelial Cell Tube Formation

Assay for studying platelet effect on angiogenesis was modified from existing protocols of endothelial cell tube formation assay [65,66,67]. Chilled liquid Matrigel (150 μL, BD Biosciences) was loaded to the wells of 48-well plate and allowed to gel at 37 ℃ for 1 h. 2H11 mouse EC (6 × 10^4^) were suspended either in 300 μL of 10% FBS/RPMI 1640 medium ±VEGF (50 ng/mL; R&D Systems, Minneapolis, MN, USA) or TCM ± TAPLT (5 × 10^7^). The cell suspensions were then added to separate Matrigel-precoated wells. The Matrigel matrix with cell culture overlay was maintained at 37 °C and 5% CO_2_ at full humidity for two hours, after which the unattached cells were removed by rinsing twice with PBS. The rinsed matrix was then incubated in PBS for imaging. Images were captured from each well by 100× magnification by inverted-phase microscopy (Axiovert 200M; Carl Zeiss Jena GmbH, Jena, Germany). The assembly of endothelial cells was assessed for tubules/networks formation of 2H11 EC from captured image. We quantified the total length and the numbers (nb) of nodes, junctions, segments, master segments, and meshes from each captured image by using Image J, the image processing program (NIH, Bethesda, MD, USA), with the Angiogenesis Analyzer plug-in tool.

### 4.10. In Vivo Matrigel Plug Angiogenesis Assay

In vivo angiogenesis assay was performed in accordance with the publicly accessible protocol of in vivo Matrigel Plug Angiogenesis Assay [66]. The ovarian cancer cell lines, MOSEC or SKOV3, were respectively mixed with its corresponding species of TAPLT into Matrigel. In brief, 1 × 10^6^ cancer cells (MOSEC or SKOV3) and 5 × 10^7^ TAPLT were resuspended in 225 μL ice-cold Matrigel (BD Biosciences, San Jose, CA, USA) at 4 °C with 25 μL ice-cold tumor-conditioned medium and 1 μL heparin (2 μg/μL) added to the mixture. Anesthesia of BALB/c nude mice were performed by intramuscular injection (IM) with 150 μL of Zoletil 50 and 2% Xylazine (1:1, *v*/*v*) before surgery, and the cell–Matrigel mixture was subcutaneously injected into the dorsal-lateral site of 8-week-old female BALB/c nude mice using an ice-cold syringe with a 24G-one inch needle. The injected mixture would form a solid subcutaneous gel plug. After 10-day implantation, mice were anesthetized with 150 μL of a mixture of Zoletil^®^ 50 (Virbac, Carros, France) and Rompun^®^ (2% Xylazine; Horse & Camel Supplies, Abu Dhabi, UAE) at a 1:1 *v*/*v* ratio, and the gelled basement membrane-like extract (BME) complex was harvested from the implanting site for examination and microscopic photography using a digital camera. In separate experiments, removed BME complexes were digested in 200 μL of Dispase solution (Collaborative Research, Bedford, MA, USA) at 37 °C overnight. Centrifugation The digested BME solution was then centrifuged (5000 rpm × 5 min, RT) to harvest the cell pellets and insoluble fractions. The resulting cell pellets were resuspended in 500 μL of 10% FBS/DMEM for 1-hr culture at 37 °C to allow cell recovery and presentation of a full profile of surface receptors. After repeated centrifuging and washing, the harvested cells were resuspended in staining solution of 200 μL of FITC-Lectin (25 μg/mL; Sigma, St. Louis, MO, USA) for 2 h incubation at RT. The stained cell pellets were again centrifuged and washed three times with cold PBS. The final pellet was resuspended in 100 μL of PBS and transferred to individual wells of 96-well plates for determination of relative fluorescence intensity by spectrofluorometry. The FITC fluorescence intensity was measured at excitation 485 nm and emission 510 nm. Calculation of FITC-Lectin concentration (ng/mL) was based on the standard curve and data used for neovascular quantitation. All assays were performed in triplicates.

### 4.11. In Vitro Tumor Cell Transendothelial Migration

Mouse 2H11 EC were grown in collagen (50 μg/mL)-coated Transwell inserts (8 μm pore size; Corning Costar) in DMEM complete medium until cell confluence was 100%. The medium was replaced with a 1:1 (*v*/*v*) mixture of TCM and RPMI 1640 medium (serum-free in upper chamber and 10% FBS in lower chamber). A cell mixture of MOSEC cancer cells (1 × 10^5^) and TAPLT (1 × 10^8^) as indicated were seeded in the upper chamber. Cancer cells were allowed to migrate for 24 h at 37 °C. The non-migrating cells were scraped off from the apical side of each insert, and cells that had migrated to the basal side of Transwell inserts were then fixed and stained using the Differential Quik Stain Kit (Modified Giemsa; EMS, Hatfield, PA, USA). After mounting the Transwell membrane on glass slides, the transmigrating cells were counted from at least three randomly captured fields by bright-field microscopy (Zeiss Axioskop; Carl Zeiss, Oberkochen, Germany).

### 4.12. Cancer Cell Colonization Assay

100 μL of TCM or TCM-suspended the indicated TAPLT (1 × 10^8^) together with MOSEC/Luc ovarian cancer cells (1 × 10^6^) were preincubated for 30 min at 37 °C and then injected to female BALB/c nude mice between 8 and 10 weeks old via tail vein in a restraint device. Mice were subjected to inhalation of anesthesia with isoflurane at 15 min, 1, 2, 3, 6 and 8 days post injection. Bioluminescence images of cancer cells colonizing in lung were taken by Xenogen IVIS200 system after 5 min of intraperitoneal (i.p.) injection with D-luciferin solution (Gold biotech., St. Louis, MO, USA). The bioluminescent signals were quantified by Living Image 3.0 (Caliper Life Sciences, Hopkinton, MA, USA).

### 4.13. ROS Assay of Indirect and Direct Cell–Cell Contact

A Transwell co-culture system with Anopore™ membrane (pore size: 0.4 μm) as the barrier was established for indirect co-culture of endothelial cells and platelets. We first seeded 1 × 10^5^ 2H11 mouse EC in culture medium of 10% FBS DMEM complete medium over collagen (50 μg/mL)-coated glass coverslip in the lower chamber (well) of a 24-Transwell plate. A 100% cell confluence of 2H11 EC monolayer was achieved after overnight culture. We briefly washed EC with 1xPBS, and changed the well medium to 1 mL of TCM containing dihydrorhodamine-123 (DHR, 30μM; Sigma, St. Louis, MO, USA). TAPLT (1 × 10^8^) as indicated in specific experiments were suspended in 200 μL of TCM and added to the top well with the membrane barrier. After 30-min indirect co-cultures, Hoechst 33342 was added to each bottom medium for another 30-min incubation, and then EC were washed, and the green-fluorescent oxidation product (rhodamine 123) derived from the intracellular reaction of DHR and H_2_O_2_, as well as the blue-fluorescence of nucleus, were captured by fluorescent microscopy. In the direct cell co-culture system, experimental conductions were similar to those described in indirect cell co-culture studies in addition to the absence of a filter for separating TAPLT from 2H11 EC.

### 4.14. Flow Cytometry Analysis of EC Surface Adhesion Proteins Induced by TCM

2H11 EC were grown on 6 cm dish until cell confluence is 100% in DMEM complete medium, and then the culture medium was refreshed with a medium having a serum concentration of 2% or lower. After 24-h serum starvation, the cell medium was changed into RPMI 1640 complete growth medium (GM), and either with the content of cytokines (TNF-α, 50 ng/mL; IFN-γ, 20 ng/mL) or TCM (50%, *v*/*v*) for another a total of 4-h cell incubation. After cell trypsinization and harvest, the cells were resuspended with flow cytometry staining buffer (eBioscience, San Diego, CA, USA), and stained with PE-conjugated specific antibodies against mouse CD51, CD106, CD162, CD54, CD31, and CD62E as well as FITC-conjugated antibody against CD62P (BD Bioscience, San Jose, CA, USA). Flow cytometry with respective isotype control antibody was also performed in parallel with this study as control experiment. The stained cells were washed, and the fluorescence intensity of PE and FITC was measured with the excitation/emission wavelength of 488 mm/575 mm and 488 mm/525 mm, respectively. A minimum of 20,000 events was collected, and data were acquired and analyzed with the ratio of CD51, CD106, CD162, CD54, CD31, CD62E, and CD62P positive cell population in total cells using flow cytometry (Cytomics FC500; Beckman Coulter, Brea, CA, USA).

### 4.15. Neutralization of VCAM-1 Binding Proteins on TAPLT Surface

Mouse TAPLT treated with a recombinant human IgG1 Fc fragment (as negative control) or recombinant VCAM-1/CD106 Fc chimera (R&D Systems, Minneapolis, MA, USA) were prepared as follows: 1 × 10^8^ TAPLT were preincubated with 440 nM of human IgG1Fc (TAPLT^IgG-Fc^) or recombinant VCAM-1/Fc chimera proteins (TAPLT^VCAM-1-Fc^) for 30 min at 37 °C, washed once with Tyrode’s buffer, and then resuspended in medium for respective experiments.

### 4.16. Cell Adhesion Assay

An amount of 1 × 10^5^ of 2H11 EC were grown in the collagen-precoated well of a 48-well microtiter plate (Thermo Fisher Scientific, Waltham, MA, USA) for 24 h until cell confluence reached 100%. After washing, the 2H11 EC monolayers were incubated in 200 μL of TCM for 2 h in a cell incubator (5% CO_2_, 37 °C) and then co-cultured with 1 × 10^8^ cell numbers of TAPLT or DsRed tagged TAPLT for 60 min of static adhesion to 2H11 EC. Non-adherent cells were removed by washing three times with PBS for 5 min each. All attached cells were fixed with 10% PBS-neutralized formaldehyde, and cells were incubated with rat anti-mouse D41 (eBioscience, San Diego, CA, USA) or mouse anti-DsRed tag antibody (Biovision, Milpitas, CA, USA) followed by incubation with goat anti-rat IgG conjugated with FITC of secondary antibody (Jackson ImmunoResearch Lab, Bar Harbor, ME, USA) or goat anti-mouse IgG conjugated with DyLight 594 of secondary antibody (Thermo Fisher Scientific, Waltham, MA, USA), respectively. 4′6-diamidin-2-phenylindol-dihydrochloride (DAPI; Roche Molecular Biochemicals, Branchburg, NJ, USA) was used for a nuclear counter stain. After sample mounting with VECTASHIELD Antifade Medium (Vector Laboratories, Burlingame, CA, USA), the adhesion of TAPLT to EC monolayer was examined by a Zeiss Axiovert 200M fluorescence microscopy.

### 4.17. Statistical Analysis

The graphic and statistical analyses were performed using the scientific graphing software Prism 5.0 (GraphPad Software, San Diego, CA, USA) and SigmaStat 3.0 (Systat Software, San Jose, CA, USA). Group average values were expressed as mean ± SEM. Unpaired Student’s *t*-test was used for comparisons between two groups. For multiple comparison of data sets that involved more than two groups, one-way analysis of variance (ANOVA) with post-hoc Dunnett’s least significant difference test was used after testing for equal variance by Bartlett’s test; ANOVA on ranks with post-hoc Dunn’s rank sum test was used if data sets were found heterogenous by Bartlett’s test. A value of *p* < 0.05 was used to define statistical significance.

## Supplementary Materials

The following supporting information can be downloaded at: https://www.mdpi.com/article/10.3390/ijms23073433/s1.
