# Supplementary material for: Thrombin-Activated Platelets Protect Vascular Endothelium against Tumor Cell Extravasation by Targeting Endothelial VCAM-1
Source: Int J Mol Sci. 2022 Mar 22;23(7):3433. doi: 10.3390/ijms23073433 (PMC8998259; doi:10.3390/ijms23073433)
Supplement: Supplementary file 1 [file ijms-23-03433-s001.zip › ijms-1613330-Supplementary Materials and Methods.pdf]

Thrombin-activated platelets protect vascular endothelium against tumor cell extravasation by targeting endothelial VCAM-1

Supplementary Materials and Methods

Flow cytometry analysis of expression of integrin and P-selectin on platelet surface

1×10^7 murine naïve platelets (PLT) or TAPLT were resuspended in flow cytometry staining buffer (eBioscience). 0.1 mL of each aliquot was placed on ice and stained for 45 min in the dark with specific antibodies respectively against mouse integrin molecules of β1(eBioscience), αD (LifeSpan BioSciences), α9 (R&D Systems) or β2 (eBioscience), as well as with CD62P-FITC antibody (BD Bioscience) against platelet activation marker of P-selectin. Isotype antibodies of corresponding species were used for control of integrin assays, and PLT without treatments of thrombin and CD62P-FITC antibody were used as negative controls (NC) of P-selectin assays. PE-conjugated secondary antibodies were used for detections of mouse integrin β1 and αD, and FITC-conjugated secondary antibodies were used for detections of integrins of α9 and β2, as well as CD62P. The stained cells were washed and resuspended with ice-cold PBS buffer. Flow cytometry was conducted using a Cytomics FC500 flow cytometer (Beckman Culter), and the fluorescence intensity of

PE and FITC were measured at the excitation/emission wavelength of 488 mm/575 mm and 488 mm/525 mm, respectively. A minimum of 20,000 events was analyzed for the ratio of integrin or CD62P positive cells to the total cell population in total cells.

Neutralization of surface-expressed αD/β2 integrin on TAPLT

TAPLT were pre-hybridized with antibodies specific for integrin monomers αD (LifeSpan BioSciences), or β2 (eBioscience). Respective antibody isotype control was also included. TAPLT (1×10^8/mL) were incubated with each antibody (20 μL/mL) in Tyrode's albumin buffer for 20 minutes at 37 °C, followed by wash with Tyrode's albumin buffer, and then re-suspended in the experimental medium for cell adhesion assay or endothelial permeability assay.

Platelet aggregation assay

Platelet counts were adjusted to $2.5\sim3\times10^8$ in 0.5 mL of Tyrode's albumin buffer containing 0.02 U/mL apyrase in small glass cuvettes for aggregation assay. The light transmission was tracked using 560CA Whole Blood Lumi Aggregometer Aggregation System. After setting stabilization of baseline for at least 1 min, the changes of light transmission was traced continuously during the entire aggregation processes after addition of either thrombin or washed TAPLT (generated by 0.01, 0.1,

or 1 U thrombin) to 0.5 mL PLT for 4 minutes, to the subsequent induction of aggregation reactivity by addition of 0.5 U thrombin and 20 μg fibrinogen.

Scanning electron microscopy

200 μL of PLT or TAPLT suspension was slowly pipetted into 800 μL of fix solution (3% glutaraldehyde and 2% paraformaldehyde in 0.1 M cacodylate buffer, pH 7.4) along the wall of the vessel wall, and fixation of the platelets was allowed to occur at 37°C for 30 minutes. Subsequently, the samples were centrifuged at 1,900 ×g for 3 minutes, the supernatant was removed and the pellet was repeatedly washed three times for 10 minutes each using SEM buffer (0.1M cacodylate buffer, pH7.4), and then pellet was resuspended in fix solution (2% osmium tetroxide in SEM buffer, pH7.4) under a chemical fume hood, and fixation on ice for 1 hour. The samples were repeatedly washed three times for 10 minutes each using SEM buffer. The fixed platelets were then serially dehydrated in ethanol at 30% v/v, 50% v/v, 70% v/v, and then 95% v/v ethanol, each concentration once at 4 °C for 10 minutes, followed by final dehydration in 95% v/v ethanol once and 100% v/v ethanol twice, each for 10 min, at 25 °C. The samples were processed by critical point drying (CPD), mounted on stub, and coated with gold in a sputter coater. The samples were observed by a HITACHI SU-8220 instrument with the beam voltage set at 5.0 kV.

Supplementary Figure Legends

(A)

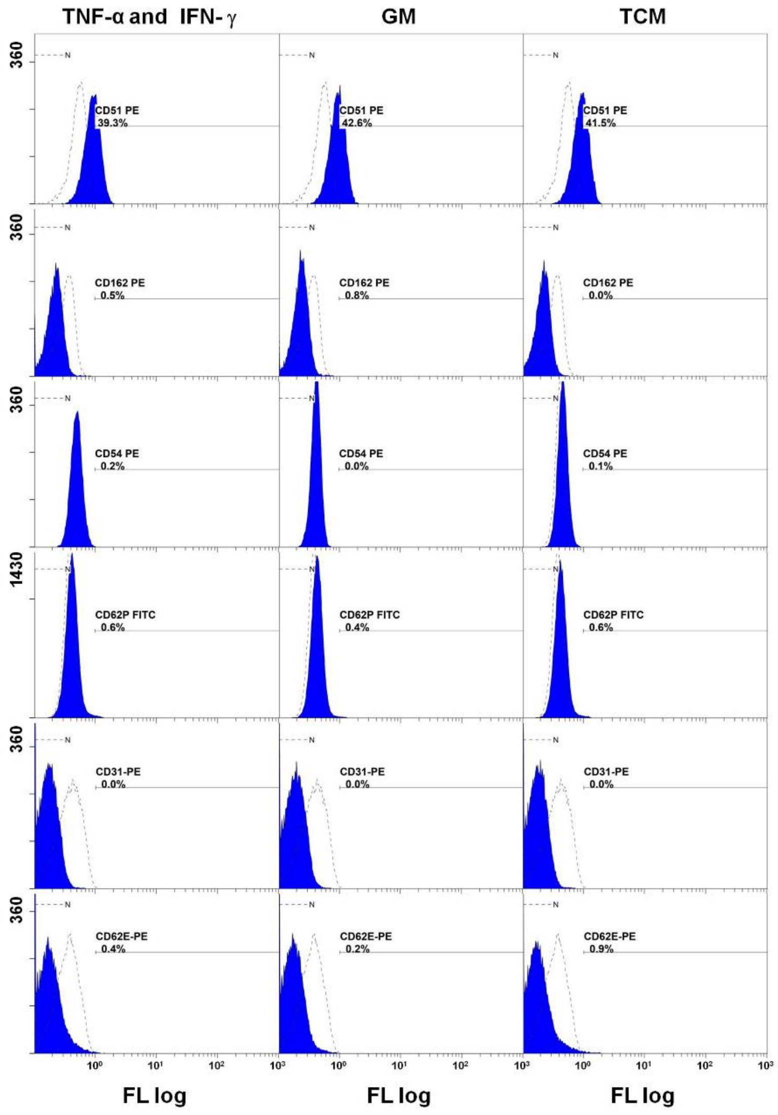

(B)

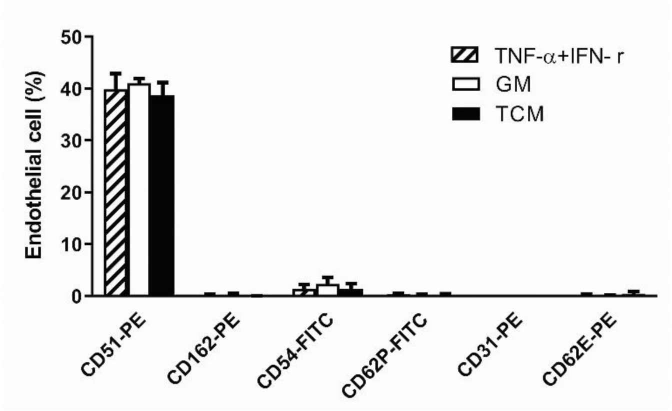

Figure S1. The adhesion protein expressions on 2H11 EC. Integrin α (CD51), PSGL-1(CD162), ICAM-1 (CD54), P-selectin (CD62P), PECAM-1(CD31), and E-selectin (CD62E) were determined by flow cytometry assay. (A) Representative flow cytometry histograms plotted by cell percentage against fluorescence intensity of immuno-stained protein (blue) or the corresponding isotype control (dashed line) on 2H11 EC. (B) Flow cytometry data were calculated from three individual experiments. Values are presented as mean ± SEM. A negative control growth medium (GM), and a positive control of GM-suspended the cytokines of TNF-α and IFN-γ also include in this study.

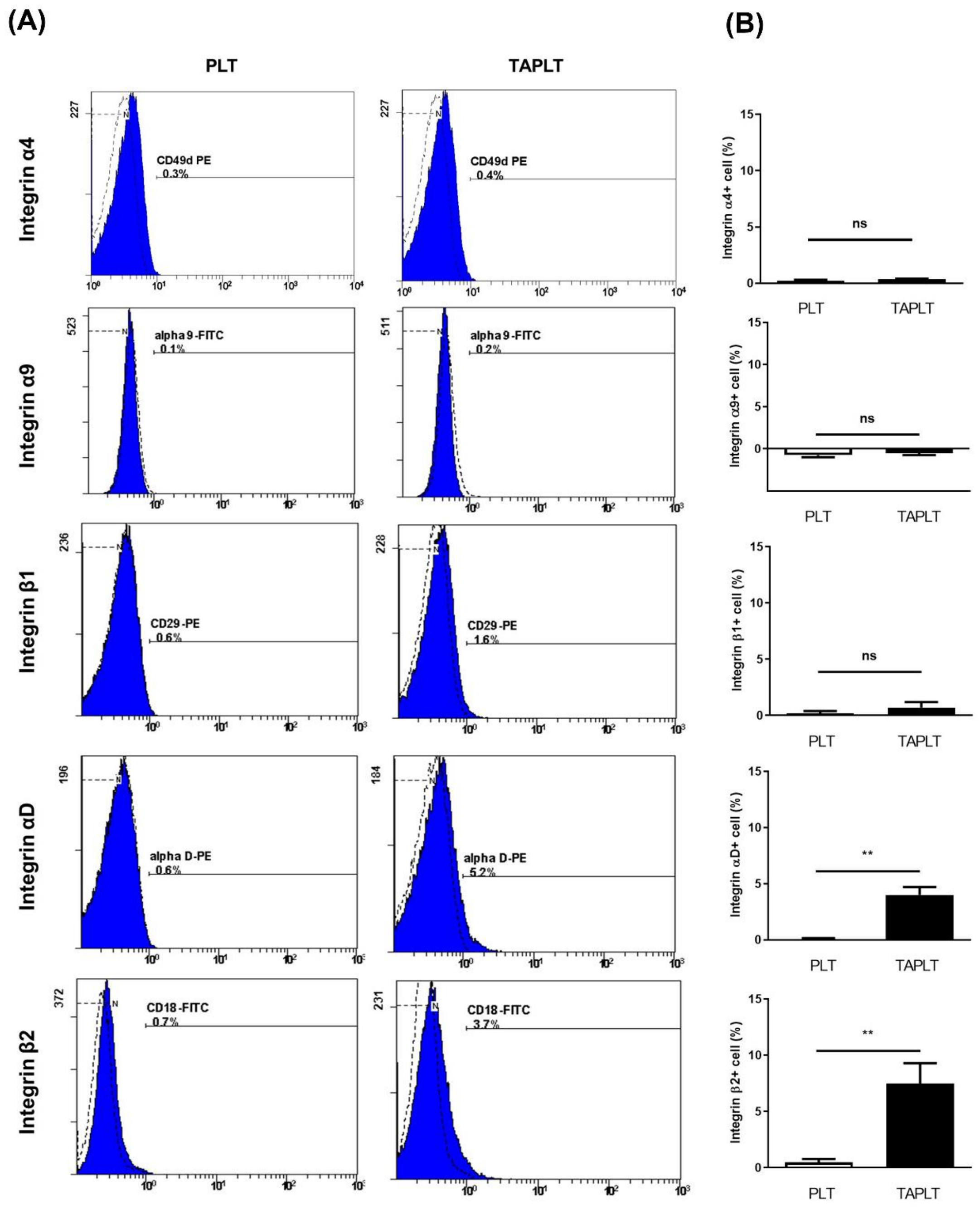

Figure S2. Integrin αD and β2 expression is predominant on surface of TAPLT. The expressions of integrin α4 (CD49d), α9, β1 (CD29), αD, or β2 (CD18) on PLT or TAPLT were determined by flow cytometry assay. (**A**) Representative flow cytometry histograms were plotted by cell percentage against fluorescence intensity of immune-stained protein. The blue histograms represent the cells with positive

expression of integrin, and the dashed lines are isotype controls. (**B**) Histogram comparison of flow cytometry results from at least two independent experiments. Values are presented as mean ± SEM; **: $p < 0.01$; ns: not significant.

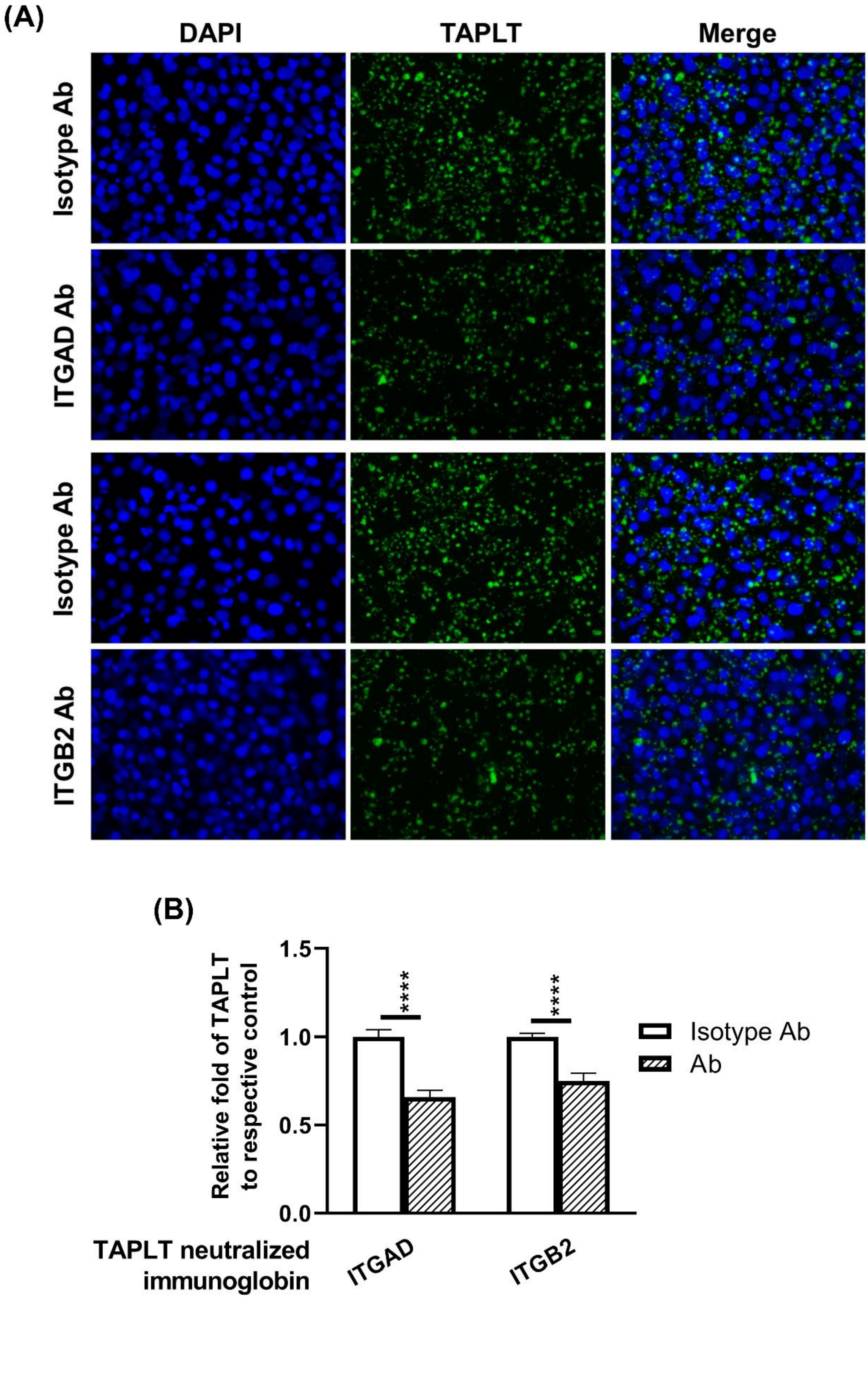

Figure S3. Neutralization of integrin αD or β2 proteins abrogates the adhesion of

TAPLT to TCM-activated 2H11 EC monolayer. TAPLT-2H11 EC monolayer co-culture was treated with neutralizing anti-αD, anti-β2 integrin, or respective isotype control antibody for 60 minutes. The attachment of TAPLT on 2H11 EC monolayer was determined by immunofluorescence assays using specific antibodies against mouse CD41. (**A**) Representative images of three independent experiments at 200× magnification were shown. (**B**) The adhered numbers of TAPLT to 2H11 EC monolayer were counted from three randomly captured fields in each sample, and data were calculated from three independent experiments using Image J software. Values are presented as mean ± SEM; ****: $p < 0.0001$. ITGAD: integrin αD; ITGB2: integrin β2.

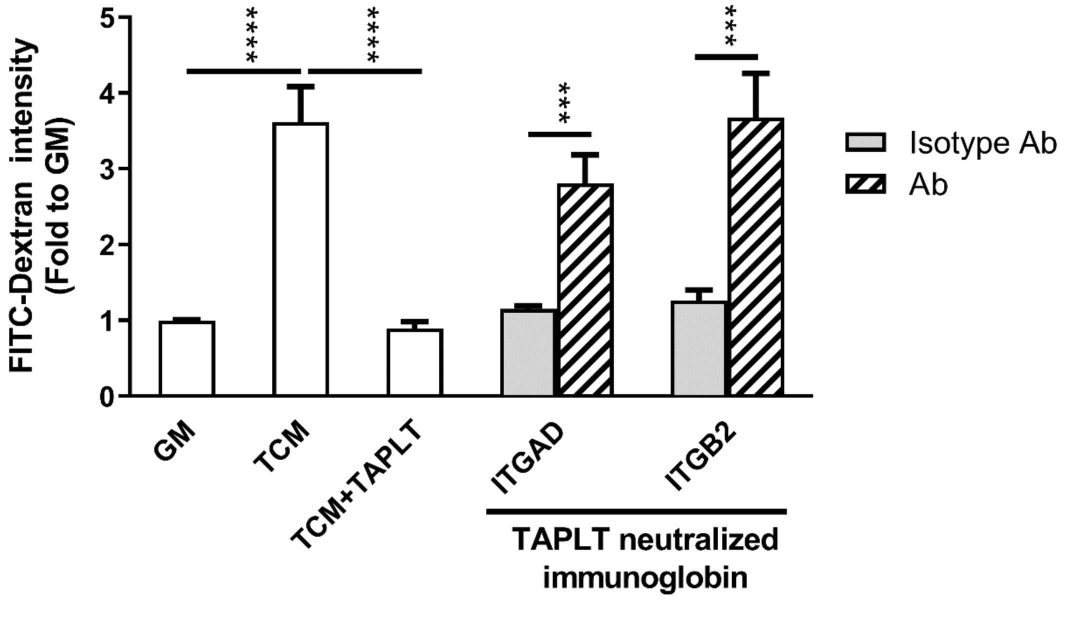

Figure S4. Neutralization of integrin αD or β2 proteins abrogates the effects of TAPLT in protecting endothelial cells against TCM-induced permeability.

TAPLT-2H11 EC monolayer co-culture was treated with neutralizing anti-αD, anti-β2 integrin, or respective isotype control antibody for 60 min in presence of TCM for endothelial permeability assay. As control, RPMI 1640 (GM) or TCM with or without TAPLT in suspension were also tested. Data were calculated from five independent experiments with a fold change to the GM group. Values are presented as mean ± SEM. ***: $p < 0.001$ and ****: $p < 0.0001$ by two-group comparison (t-test); ITGAD: integrin αD; ITGB2: integrin β2.

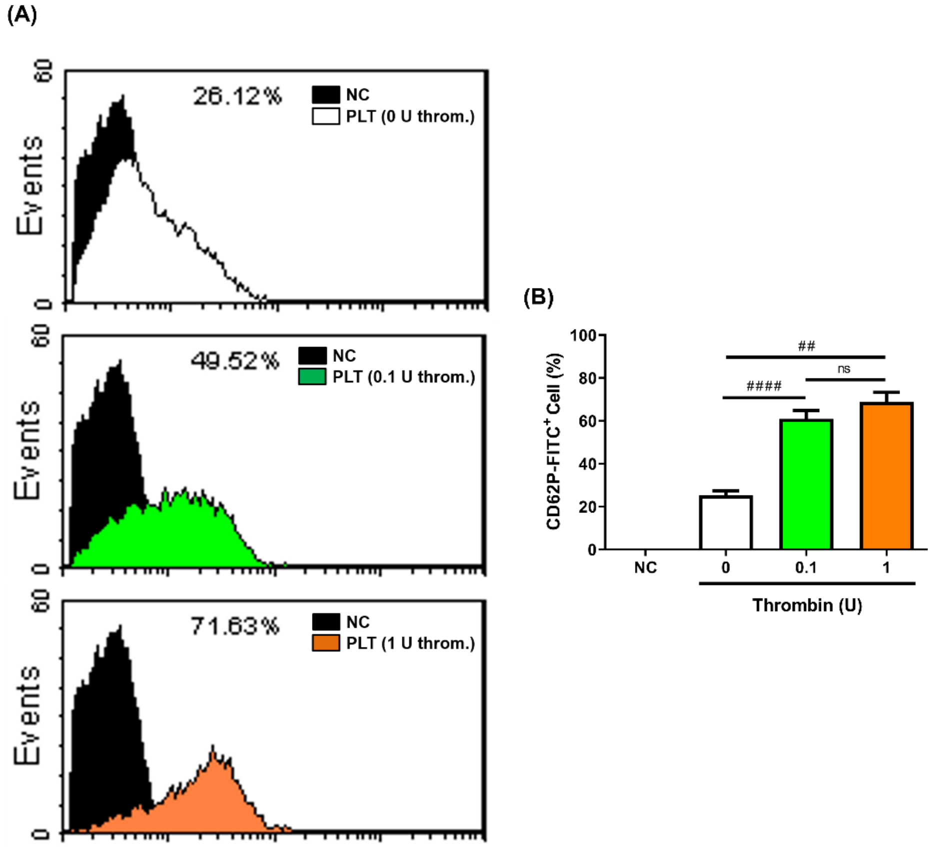

Figure S5. Expression of platelet activation marker CD62P. Murine platelets were

activated with thrombin (0, 0.1, or 1 U). CD62P expression on platelets were determined by flow cytometry. (A) Representative flow cytometry histograms of CD62P expression (white, green, orange peak) versus the corresponding negative control (black peak) on murine PLT. (B) Percentages of C62P+ platelet were compared in at least four individual experiments. Values are presented as mean ± SEM. ##: $p < 0.01$ and ####: $p < 0.0001$ by multiple comparisons (ANOVA); ns: not significant; NC: negative control (untreated and unstained platelets).

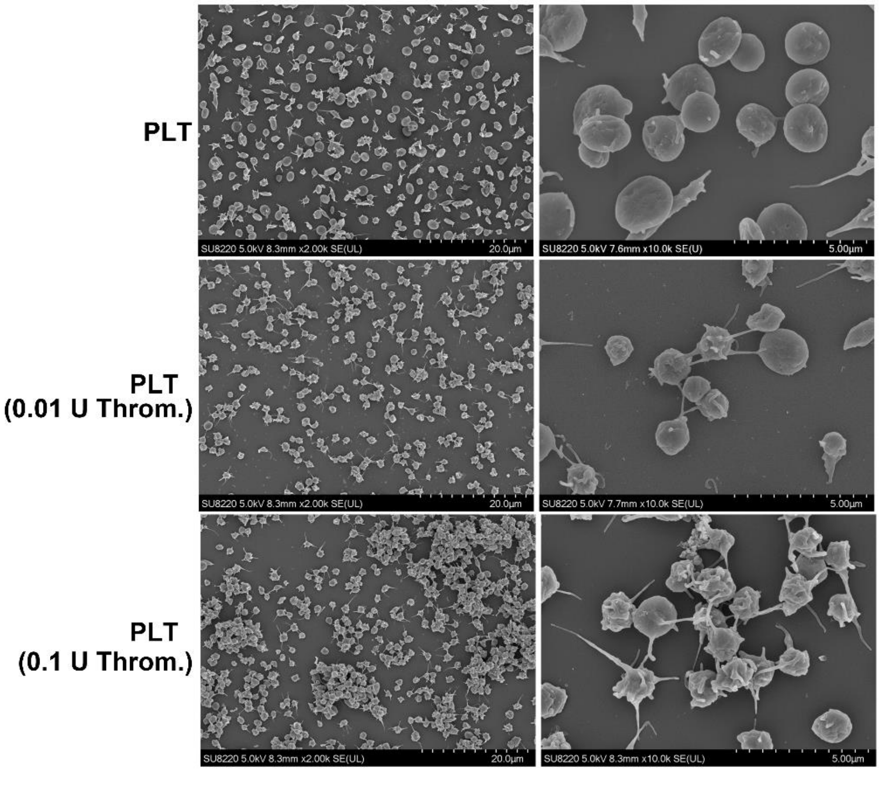

Figure S6. Morphological observations of thrombin-activated platelet. Murine platelets were activated with thrombin (0, 0.01, 0.1 U), and observed by SEM. SEM

images were acquired using the HITACHI SU-8220 at electron voltages of 2 keV (left panel) and 10 keV (right panel). Throm: Trombin.

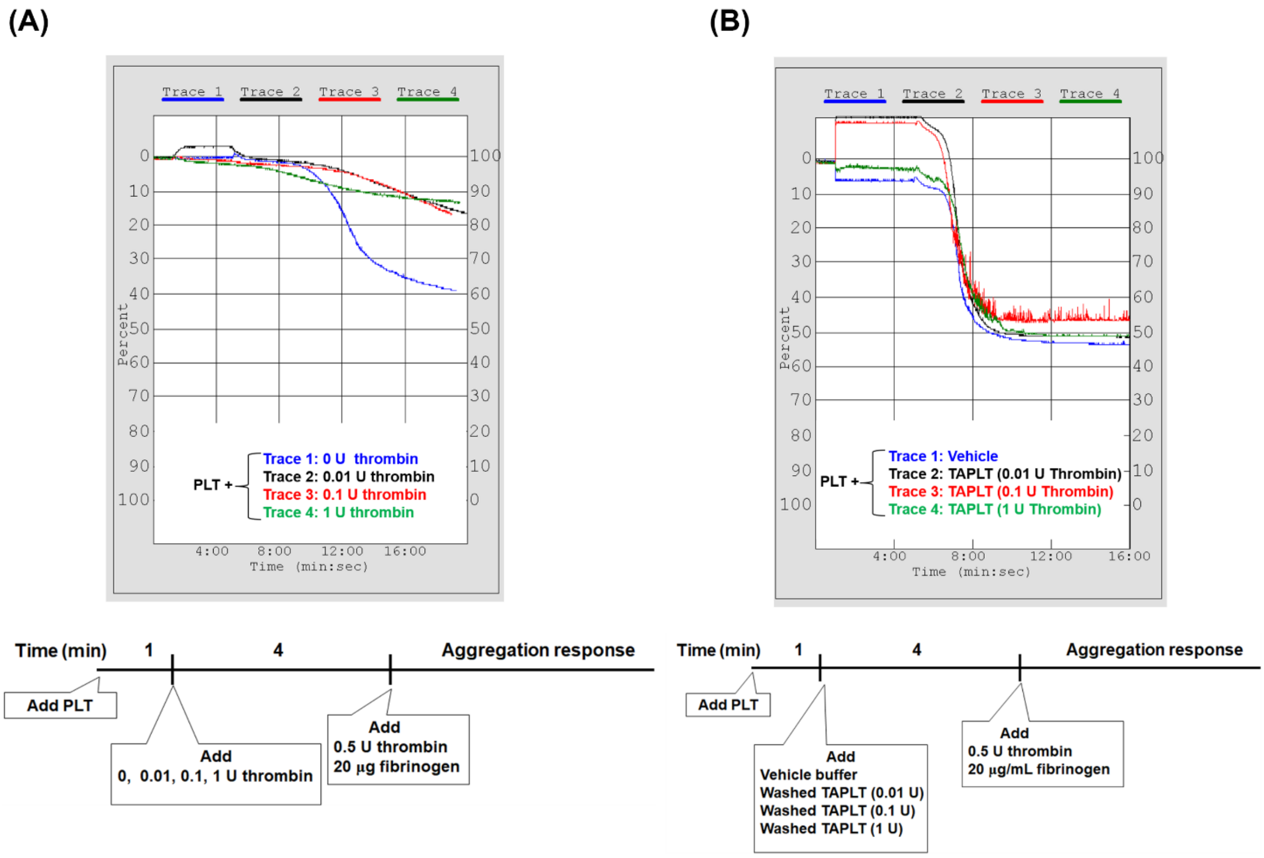

Figure S7. The absence of aggregative activity of TAPLT has little impact on the aggregation responses of PLT. (**A**) The aggregation response of TAPLT derived from 0.01, 0.1, or 1 U thrombin treatment was measured. Representative aggregation curves of three independent experiments were shown in top panel with a percentage of light transmission. Schematic representation of the timescale of the addition of the PLT, thrombin agonist, and thrombin/fibrinogen constituted coagulation factors is shown in bottom panel. (**B**) The aggregation response of PLT mixed with TAPLT

derived from 0.01, 0.1, or 1 U thrombin treatment was measured. Representative aggregation curves of three independent experiments are shown in top panel with a percentage of light transmission. Schematic representation of the timescale of the addition of the PLT, distinct activity of washed TAPLT, and thrombin/fibrinogen constituted coagulation factors is shown in bottom panel.
